# An Academic Relative Value Unit System: Do Transparency, Consensus, and Accountability Work?

**DOI:** 10.5811/westjem.2019.8.43832

**Published:** 2019-10-14

**Authors:** Kristin A. Carmody

**Affiliations:** New York University School of Medicine, Ronald O. Perelman Department of Emergency Medicine, New York, New York

## Abstract

**Introduction:**

Academic medicine continues to struggle in its efforts to compensate scholarly productivity. Academic achievements receive less recognition compared to clinical work, evidenced by a lack of reduced clinical hours or financial incentive. Core departmental education responsibilities are often distributed inequitably across academic departments. An approach using an incentive program, which emphasizes transparency, equity, and consensus may help academic departments share core education responsibilities and reward scholarly activity.

**Methods:**

We launched a two-stage approach to confront the inequitable distribution of educational responsibilities and to recognize the scholarly work among our faculty. In the first stage, baseline education expectations were implemented for all faculty members, which included accountability procedures tied to a financial incentive. The second stage involved the creation of an aAcademic rRelative vValue uUnit (ARVU) system which contained additional activities that were derived and weighted based on stakeholder consensus. The points earned in the ARVU system were applied towards additional financial incentive at academic year-end. We compared education contributions before and after implementation as well as total points earned in the ARVU system.

**Results:**

In the first year of implementing education expectations, 87% of faculty fulfilled requirements. Those with a heavier clinical load made up the majority of deficient faculty. Those who did not meet education expectations were notified and had their year-end incentive reduced to reflect this. Faculty conference attendance increased by 21% (P<.001) and the number of resident assessments completed increased by 30% (P<.001) compared to the previous year. To date, faculty across the department have logged a total of 1,240 academic activities in the database, which will be converted into financial bonus amounts at year-end.

**Conclusion:**

We have seen significant increases in faculty participation in educational activities and learner assessments as well as documentation of activities in the ARVU system. A similar system using different specialty-specific activities may be generalizable and employed at other institutions.

## INTRODUCTION

Academic medicine faces a challenge on how to balance the objectives of revenue production with compensation of scholarly achievement. Historically, “relative value units” have been used to incentivize physicians to improve clinical productivity, but these systems have neglected to recognize non-clinical achievements, such as those related to teaching, academic leadership roles, or other scholarly activity. Many non-clinical activities do not earn a reduction in clinical hours or financial incentive, which may result in decreased motivation to contribute academically as well as frustration and burnout. As faculty members work to advance in their professional careers, diminished scholarly output may create a barrier for promotion possibilities at traditional academic institutions. All of this may result in less time devoted to teaching and diminished opportunities for mentorship and role modeling for learners.

To foster academic productivity and the retention of talented physicians, academic medicine must recognize and reward the effort that is necessary to thrive within it.^1^ Models have been introduced over the past decade that focus on incentivizing non-clinical activities. Some of these models have focused solely on education and teaching commitments using a teaching or educational value unit system to weigh activities.^2^–^5^ Others have cast a broader net encompassing all academic activities, including education, teaching, committee and administrative roles, and research, using a clinical or academic relative value unit (ARVU) model.^6^–^8^

Problems were identified in our department with regards to education and scholarly activity. The residency group and a small group of core faculty have traditionally carried much of the teaching effort, resulting in an unequal distribution of educational commitments across the department. In addition to education, many in the department participate in other scholarly work such as research projects earning grant funding, peer-previewed publications, lecturing engagements, and leadership or committee positions. Similar to other academic institutions, our department has experienced difficulty tracking faculty activities outside of clinical work. Faculty frustration has resulted from many of these activities not being compensated financially or rewarded with reduced clinical hours. Furthermore, junior faculty lacked an understanding of the importance of tracking academic activities as a way to monitor their progress and to focus on areas that required more attention in preparation for the promotion process.

We brainstormed ideas regarding how to expand faculty commitment to better align with our academic mission, to prepare faculty for promotion, and to create an improved infrastructure fostering resident and student mentoring. Our project had several objectives: 1) realign and redistribute the responsibility for meeting education needs equitably across the department; 2) create a system of accountability and transparency based on faculty consensus; 3) recognize and reward academic activities that go above minimum expectations; 4) align faculty academic productivity with institutional promotion procedures; 5) build a system that houses academic activities in a format consistent with institutional teaching portfolio expectations; 6) incentivize and increase departmental scholarly output; and 7) build a system capable of supporting an academic mentoring infrastructure for our learners.

In 2017 we initiated a two-stage project to redesign education expectations and to identify and recognize the full spectrum of academic activities among all faculty. Stage one involved the creation of a mandatory baseline educational participation process; stage two, implemented later, involved the creation of an ARVU points system with identified voluntary academic participation. Both stages of the project were tied to an academic financial incentive awarded at year-end. Our goal was to determine the effects of this project on faculty baseline participation in educational activities as well as monitor academic productivity and advancement within the department.

## METHODS

### Study Design and Setting

Institutional review board approval was not sought for this project because it was conducted for quality improvement purposes.

### Methods and Measurements

#### Stage One: Baseline Education Expectations

Stage one, initiated in July 2017, created minimum education expectations and accountability procedures, incorporating two related requirements. The first included attending a minimum number of resident conferences per year, inversely proportional to a faculty member’s clinical load. The second element required participation in a module system, created by the residency, where each month represented one module (12 in total throughout the year) and focused on a particular topic. Each faculty member was required to sign up for and commit to specific dates during a module where they were responsible for taking part in teaching activities assigned by the residency or undergraduate medical education group. These activities included such things as giving a lecture, moderating a journal club, running a small group session, or teaching a procedural skills lab among others. The sign-up process afforded some flexibility and choice, as faculty could pick dates that worked for them and topics they were most interested in. Conference attendance required only the presence of faculty in the audience, but module participation required the active participation of faculty in specified activities.

Conference attendance and module participation were chosen as minimum expectations for two reasons: firstly, all faculty historically have been expected to participate in residency and student teaching as part of their academic appointment to the medical school; and secondly, these activities were considered to require the heaviest lift and were inequitably distributed among the faculty. These new expectations were required of faculty across the department and were tied to a newly created academic incentive awarded at fiscal year-end. The faculty who did not meet these new education expectations were not eligible for this financial incentive.

After soliciting feedback on these new expectations through faculty meeting discussions and offline conversations, most agreed that the new expectations were not overly burdensome. However, two main concerns surfaced. One was that the academic incentive was not reflective of other non-clinical activities valuable to the department’s mission. A second concern brought forth by the residency leadership was that the expectations did not include resident assessments, which historically had a low response rate. Based on this feedback, the baseline education expectations were revised to include completion of a percentage of resident post-shift assessments over the academic year, inversely proportional to a faculty member’s clinical load. During the first year (academic year [AY] 2017–18), the requirements included only conference and module participation. The residency assessment requirement was subsequently enacted in the following year (AY 2018–19). Table 1 lists the final baseline education expectations required of faculty members. Before employing these education requirements, all faculty members were notified of the consequences of not fulfilling expectations, which included ineligibility for any academic incentive and an inability to participate in the voluntary ARVU system.

#### Stage Two: Academic Relative Value Unit System

In May 2018, stage two began, which involved the creation of an ARVU system to encompass all other academic activities. It was decided that the ARVU system would be voluntary, but to participate the baseline education expectations outlined in stage one had to be fulfilled. For the first step of this stage, the vice chair for education created a list of preliminary activities to be included in the ARVU system, such as teaching, lecturing, publications, grants, committee memberships, and leadership positions. These additional activities were ones in which faculty were already participating that aligned with the academic mission of the department, but had not been captured within the baseline education expectations, did not earn clinical hours reduction from the department or institution, or were not an implicit part of a faculty member’s role based on his or her leadership position. The thought was that activities that earned a clinical reduction in hours were already being financially rewarded, and this system was designed to recognize activities not yet distinguished. An example includes fellowship activities, which were not included because fellowship directors have a reduction in clinical hours to support their leadership role.

After the initial list was assembled, it was shared with a select group of 11 leaders within the department, including residency leadership, undergraduate medical education leadership, fellowship directors, the research division, and the pediatric emergency medicine division. The participants were selected due to their various leadership roles in the department, their dedication to scholarly achievement in their own careers, and the high priority they placed on these activities within their respective divisions. These qualifications placed these faculty members in a prime position to help generate a comprehensive list of activities relevant to each division. After multiple discussions and written communications using a modified Delphi method, the group reached consensus on the activities that were to be included.

The unique part of this project was the third step, which included a survey that was created and analyzed using Qualtrics software (Provo, UT, and Seattle, WA) and distributed to a group of 60 faculty members across the department. These faculty members were chosen out of a total of 123 because they were identified as department members who regularly participated in the activity list created by the leadership group. Because these faculty members were the most active in these activities, they were in the best position to review the list and evaluate each activity to its fullest. Furthermore, because it was decided that the ARVU system would be voluntary, they were deemed the faculty most likely to be invested in and use this new system. Finally, one of the goals of this mission was to get faculty buy-in as they were the most important stakeholders in this endeavor, and this was achieved by allowing them a voice and to feel empowered in the final steps of this project.

The survey included all agreed-upon activities and asked faculty to rate each on a scale from one (minimal effort) to four (most effort) (Appendix 1). A short description of the activity in question was included to help faculty decide on the point values assigned. The 11 faculty members who contributed to the final list of activities created these descriptions. Effort was defined by the time needed to commit or prepare for a particular activity, the ongoing effort needed to sustain the activity if it involved a longer commitment than just one session, and whether the activity required a passive presence or more active participation. For example, activities that required a sustained effort included such things as grant involvement, committee membership, or a leadership position.

As expected, some subjectivity was involved in the voting for various reasons, such as the activity being one in which the responsive faculty member participated in himself or herself, or differing opinions regarding how much preparation time might be needed for such things as a lecture. To help reduce this bias, the survey was sent to many faculty members with different roles and responsibilities to obtain a consensus and to dilute idiosyncratic points of view. Furthermore, the knowledge of and dedication to each activity that the chosen faculty members had and the descriptions provided helped to further reduce bias in the points system. The survey also included free-text fields where faculty could input additional activities that they felt should be added to the list.

Of the 60 faculty members surveyed, 49 (82%) responded and completed the survey in its entirety. The activities, ranked from highest to lowest based on the mean score including standard deviations, are presented in Table 2. The standard deviation was less than one for all activities included in the survey. The mean of each activity was translated into final points to be awarded in the ARVU system. Activities with higher means earned more points. Any activities that were similar in description and mean score were assigned the same number of final points. We introduced the final list and point system at a faculty meeting prior to implementing, and after this final feedback round, we launched the system in December 2018. The free-text responses were also reviewed, and these activities were added to the list and also voted on by the faculty group to create the final list with points.

**Figure 1:**
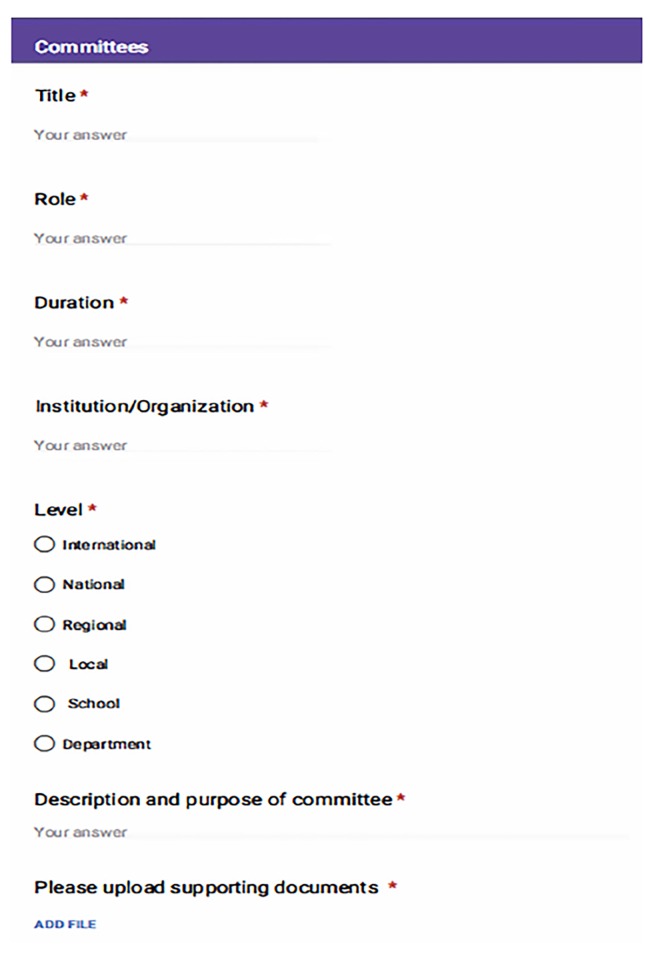
Google document used to document faculty’s academic relative value unit activities.

The next steps for the project included creating a database where faculty could log their completed activities. We created a Google form that listed all activities in the ARVU system where faculty members could select the activity in which they participated (Figure 1). Each activity had an associated dropdown menu that asked for additional information, such as title, date, location, description, proof of activity, and an ability to upload documents. We then created a dashboard in the analytics platform Tableau (Seattle, WA), containing all activities. Statistics for the baseline educational expectations (conference attendance, module participation, and resident assessments) automatically loaded into the dashboard and could not be edited by faculty members.

**Figure 2:**
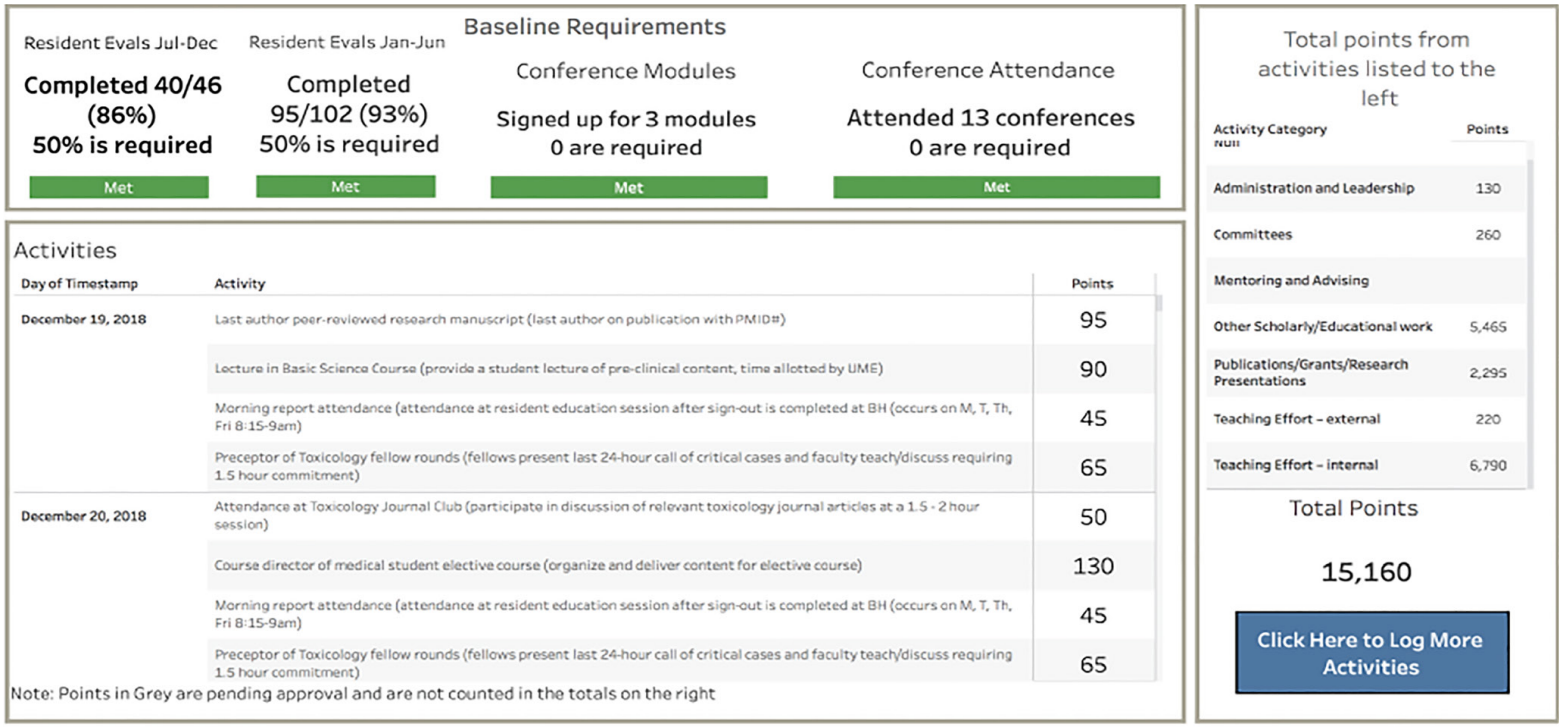
Dashboard with education expectations, academic relative value unit activities, points, and portfolio categories.

The ARVU activities logged into the Google form also fed directly into the dashboard for display. The full dashboard displayed each faculty member’s baseline education expectations, whether they had met requirements, the activities that they had entered into the ARVU point system, and total points earned to date (Figure 2). Final points were earned after academic leadership reviewed, approved, and signed off on each submitted activity. Each month, the system automatically e-mailed a link to each individual’s dashboard notifying faculty how many points they had earned to date and of any participation deficiencies.

The medical school requires a teaching portfolio for faculty seeking promotion on the scholar track. This portfolio requires faculty to document their achievements in the following categories: teaching effort, mentoring and advising, administration and leadership, committees, and teaching awards. All ARVU activities were reviewed and categorized based on the elements of the teaching portfolio. These activities not only show up as itemized items with points, but they are also grouped into the appropriate portfolio category and are displayed on each individual faculty member’s dashboard. This allowed each faculty member to see how much scholarship they had completed within each of the teaching portfolio categories and in which areas they were lacking that deserved more attention. This provided faculty with a readily accessible repository of activities that could be transferred directly into the correct category of their teaching portfolio, facilitating tracking of activities upon which one needed to focus for promotion.

### Outcomes and Analysis

Compliance with baseline education expectations was determined by evaluating each individual faculty member’s conference attendance, module participation and completion of resident assessments. We evaluated the effect of the expectations on conference attendance and resident assessments by using paired t-tests performed in Microsoft Excel 2016 and by tracking individual faculty member’s compliance pre- and post-implementation. Faculty who were absent for prolonged periods of time and new faculty were not included in the analysis. The ARVU system was tracked since implementation to determine number and type of activities logged.

## RESULTS

A total of 123 faculty members were expected to participate in the baseline education expectations. At the end of the academic year in June 2018, 107 faculty (87%) had met requirements. Failure was defined as not attending the required number of conferences per year or not participating in the module system. Of the 16 who did not meet expectations, 94% signed up for conference modules to participate in specific activities, but none of them met overall required conference attendance. Of the deficient faculty, five worked full time at 28 or fewer hours, 10 were full time at more than 28 hours, and one was part time. Those who did not meet education expectations were notified and had their year-end AY 2017–18 financial incentive reduced to reflect this deficiency.

We compared an individual faculty member’s conference attendance in AY 2016–17 and AY 2017–18 to determine any changes after implementing the new expectations. Overall, faculty attended 21% (P<.001) more conference days after expectations were implemented compared to the prior year. Preliminary data for the following AY 2018–19 reveals that conference attendance increased by 15% (p = .096). The number of resident assessments completed in AY 2017–18 among all faculty was 2837 compared to preliminary AY 2018–19 assessments of 4049, resulting in a 30% (p<.001) increase since expectations went into effect.

To date, faculty across the department have logged a total of 1240 academic activities in the database. The distribution of points across categories is highlighted in Table 3 with most points earned through teaching activities at the medical school or through other scholarly work that doesn’t necessarily fit into the other categories of the teaching portfolio. Leadership will review each faculty member’s individual records to determine if they have met baseline education expectations. The faculty who meet expectations will receive the set baseline incentive and have the potential to earn more financial incentive based on the number of points they have earned in the ARVU system. Once all the data is analyzed, the points will be converted into financial bonus amounts based on the number of faculty who are eligible and the amount of funds available.

## DISCUSSION

This project has resulted in preliminary positive effects on both education and documentation of scholarly work within our department. The first stage resulted in an overall increase in conference attendance and participation even prior to implementing the ARVU system. It is possible that these positive findings were a result of the academic incentive being dependent on meeting education expectations. However, in offline discussions with multiple faculty members, it appears that there was a shame factor that also contributed to improved attendance. Multiple faculty expressed their relief that many were being called out on their low attendance and participation and that faculty who had historically carried much of the teaching responsibility were now being recognized. In the same vein, resident assessments increased in the second year by a considerable amount, without any other changes being made to the system, and therefore were likely a result of the new expectations. The increase in assessments does not necessarily mean better quality, and this will need to be evaluated going forward to determine full impact. The improved participation in educational activities as a result of financial incentives or other measures is consistent with reports from other institutions and existing literature.

There is a clear correlation between faculty documentation of scholarly output and the ARVU system, as there was no system in place prior that allowed tracking of activities. The increase in activities and documentation will need to be followed from year to year to draw conclusions on overall scholarly activity among individual faculty members and throughout the department. Unlike previous literature describing ARVU systems, our project has emphasized the ability to house activities in one place that can be transferred into a faculty member’s teaching portfolio, thereby further incentivizing the use of this system outside of financial rewards.

We will continue to track baseline education expectations and the ARVU system across the department as well as continuously seek feedback from faculty and make changes as needed. This process will continue to be refined over time based on faculty feedback and departmental and institutional priorities. The majority of faculty who did not qualify for the academic bonus last year worked more than 28 clinical hours per week, and thus time issues may have affected compliance. To further probe this finding and facilitate educational commitments, we will solicit additional feedback from this group of faculty members to explore participation barriers that may be addressed in the future.

We hope to follow the scholarly output of the department over time using the ARVU system as an estimate of faculty productivity. Our longer-term goals will be to see the effects of this system on the promotion process within the department with an expectation that more junior faculty will become eligible for advancement. These effects will be evaluated by tracking the progress and content of junior faculty teaching portfolios compared to previous years and time to successful promotion. With a bottom-heavy young faculty group, our expectation is that this system will better prepare people for promotion as they can track their activities and determine where they need to place more effort to enhance their portfolio. Finally, this system will be used to improve the mentorship infrastructure within the department. Assigned faculty mentors will use the ARVU dashboard to mentor junior faculty on their progress for promotion. This dashboard will provide another data point for mentors to advise junior faculty where they need to focus their efforts in order to progress professionally.

## LIMITATIONS

There was likely subjectivity and bias in faculty assigning points to activities based on effort. Faculty may have ranked certain activities higher than others due to their own participation in the activity in question. In addition, faculty have different opinions on what type of effort may go into an activity; for example, a lecture may be easily prepared by some and take a lot of effort for others. We attempted to remove some of this subjectivity and bias by including faculty in this process who are the most committed to academics in our department. Many of these faculty participate in these activities on a regular basis and, therefore, we believed they were most committed to creating a fair transparent system to reward achievements. Furthermore, the standard deviation for each activity was not large enough to have created significant discrepancies in where a particular activity was ranked.

This was a project initiated at a single site, which may limit its generalizability to other institutions. However, similar methods could be used to create site-specific prioritized activities that may enhance its use at other institutions. Finally, it is possible that the increase in conference attendance and resident assessments was confounded by other factors. The changes could have been simply due to faculty feeling the need to attend more conferences or better evaluate our learners, but the effects coinciding with the implementation of new expectations is unlikely to be coincidental.

## CONCLUSION

Although other institutions in a similar fashion have developed ARVU systems, using consensus-type methods, none of these systems have engaged a large faculty group to rank activities and assign final points. The methods we used to derive this system were iterative, transparent, and collaborative. This process was unique because it included multiple faculty stakeholders who had different roles and priorities within our department to create the system. The selected activities were inclusive and respectful of all efforts.

We have already seen significant increases in faculty participation in learner teaching activities and assessments. In addition, for the first time in the department’s history, we have taken steps to recognize all of the other academic activities that don’t receive funding or reduced clinical time. A similar system, using the same methods outlined above, but with different specialty-specific activities, may be generalizable and employed at other institutions.

## Supplementary Information



## Figures and Tables

**Table 1:** Baseline education expectations for faculty.

Faculty category	Conference attendance	Module requirement	Resident post-shift assessment completion
Full time ≤ 28 hours	10 conferences/year	2 modules/year (2 months)	75%
Full time > 28 hours	5 conferences/year	1 module/year (1 month)	50%
Overnight	5 conferences/year	1 module/year (1 month)	50%
Part time or non-ACGME fellow	5 conferences/year	1 module/year (1 month)	50%

*ACGME*, Accreditation Council for Graduate Medical Education.

**Table 2:** Final academic relative value unit activities with mean points and standard deviations.

Activity	Mean	Standard Deviation
Principal investigator (PI) on federal grant	3.80	0.64
Principal investigator (PI) on foundation grant	3.71	0.67
Principal investigator (PI) on industry grant	3.65	0.69
Course director of medical student selective course	3.57	0.70
First author peer-reviewed research manuscript	3.55	0.70
Primary textbook editor	3.53	0.79
Course director of medical student elective course	3.49	0.70
Principal investigator (PI) on internal school grant	3.49	0.79
Principal investigator (PI) on internal Department of EM grant	3.43	0.81
Residency module leader	3.33	0.77
Leader Scholarly Academy	3.27	0.80
Co-investigator on federal grant	3.27	0.72
Textbook chapter	3.27	0.72
Lecture at international, national or regional meeting	3.24	0.72
Co-investigator on foundation grant	3.18	0.72
Grand rounds lecture - external institution	3.16	0.71
First author non-research manuscript	3.16	0.68
Journal editor	3.14	0.78
Chair of national/regional education committee	3.12	0.72
Grand rounds lecture - internal	3.08	0.78
Lecture at resident conference	3.00	0.53
Lecture at PEM conference	3.00	0.57
Outside lecture or teaching session at another teaching institution	2.98	0.71
Leader at faculty development session	2.94	0.79
Content creator and/or editor of educational site/blog/podcast faculty	2.92	0.85
Participant/mentor CPC	2.86	0.78
Mentor resident scholarly project	2.86	0.78
Abstract presenter national meeting	2.80	0.64
Last author peer-reviewed research manuscript	2.78	0.89
Co-author peer-reviewed research manuscript	2.73	0.69
Abstract presenter regional/local meeting	2.71	0.67
Mentor of Medical Student International Health Program	2.65	0.87
Lecture at fellow core curriculum session	2.65	0.69
Preceptor of Medical Student Scholarly, Research Concentration	2.61	0.72
Project lecture in basic science course	2.59	0.81
Lecture to other NYU residents, faculty, students or staff	2.57	0.70
Journal reviewer	2.57	0.76
Last author non-research manuscript	2.53	0.81
Co-author non-research manuscript	2.53	0.70
First author on case report	2.53	0.64
Member of Residency Program Evaluation Committee	2.47	0.93
Preceptor for the Patient Longitudinal Ambulatory Clinical Experience	2.43	0.73
PEM joint conference liaison	2.43	0.64
Residency interviewer	2.39	0.88
Lecture at Toxicology Rotators Conference	2.39	0.69
Journal club moderator at resident conference	2.37	0.63
Lecture in medical school course for elective or selective	2.37	0.77
Member of NYUSoM educational committee	2.37	0.80
Co-author abstract national meeting	2.33	0.71
Co-author abstract regional meeting	2.31	0.77
Primary URiM Summer Fellowship Student faculty mentor	2.29	0.76
Preceptor morbidity and mortality conference	2.27	0.69
Member of Clinical Competency Committee	2.27	0.69
PEM conference journal club moderator	2.27	0.60
PEM journal update presenter	2.27	0.60
Member of national/regional education committee	2.27	0.69
Instructor at resident procedure, simulation or multi-modal workshop	2.24	0.69
Participant URiM Summer Fellowship	2.24	0.74
Instructor Inter-clerkship intensive (ICI) courses	2.24	0.66
Instructor in Practice of Medicine	2.24	0.72
Leader in-situ simulation session	2.22	0.65
Instructor medical student ultrasound workshops	2.20	0.67
Commentary/letter to editor	2.20	0.70
Co-author case report	2.20	0.61
Preceptor/Participant Sonolympics	2.18	0.80
EM Foundations Curriculum Faculty Facilitator	2.18	0.80
Instructor Transition to Residency Course	2.16	0.58
Instructor ATLS	2.14	0.81
Participation in First Night on Call for Interns	2.14	0.67
Preceptor/Participant EM Olympics	2.12	0.82
Instructor at PEM procedure or simulation workshop	2.10	0.61
Preceptor of toxicology bedside rounds	2.08	0.75
Primary medical student faculty mentor	2.06	0.65
PEP talks to students	2.04	0.67
Primary mentor on resident lecture	2.02	0.65
Small groups facilitator at resident conference	2.02	0.68
Preceptor of toxicology fellow rounds	2.02	0.71
Medical school interviews	2.00	0.70
Participation in oral boards preparation	1.98	0.59
PALS instructor	1.98	0.65
Ultrasound scanning shifts with residents	1.96	0.60
Ultrasound scanning shifts with medical students	1.96	0.60
Participate in NYCPCC afternoon rounds	1.94	0.79
Instructor in student simulation or workshop sessions	1.88	0.59
Participation in Emergency Medicine Interest Group	1.86	0.61
Preceptor of medical student ultrasound OSCE	1.80	0.61
Medical student case session for EM selective or elective	1.76	0.77
Participation in Standardized Direct Observation Assessment Tool	1.61	0.66
Participation in EM/PEM conference	1.59	0.67
Attendance at Ultrasound Conference	1.49	0.61
Attendance at Education Journal Club	1.47	0.58
Attendance at Toxicology Journal Club	1.43	0.57
Attendance at PSQI Journal Club	1.41	0.57
Attendance at Scholarly Academy	1.35	0.52
Attendance at Toxicology Consultants’ Conference	1.31	0.54
Resident advisor	1.30	0.51
Attendance at faculty development session	1.29	0.49
Morning report attendance	1.22	0.46

*EM*, emergency medicine; *PEM*, pediatric emergency medicine; *NYU*, New York University.

*NYUSoM*, New York University School of Medicine; *URiM*, Underrepresented Minorities in Medicine; *PEM*, pediatric emergency medicine; *ATLS*, Advanced Trauma Life Support; *EM*, emergency medicine; *PEP*, The Prevention and Education Partnership; *PALS*, Pediatric Advanced Life Support; *NYCPCC*, New York City Poision Control Center; *OSCE*, Objective Structured Clinical Exam.

*EM*, emergency medicine; *PEM*, pediatric emergency medicine; *PSQI*, Patient Safety and Quality Improvement.

**Table 3:** Total activities, points, and categories logged to date.

Activity Category	Points
Administration and leadership	260
Awards	180
Committees	825
Mentoring and advising	755
Other scholarly/educational work	29,050
Publications/grants/research presentations	5,355
Teaching effort - external	2,245
Teaching effort - internal	22,500
Total points	61,170
