# Supplementary material for: An Academic Relative Value Unit System: Do Transparency, Consensus, and Accountability Work?
Source: West J Emerg Med. 2019 Oct 14;20(6):939–47. doi: 10.5811/westjem.2019.8.43832 (PMC6860393; doi:10.5811/westjem.2019.8.43832)
Supplement: Supplementary file 1 [file wjem-20-939-s001.pdf]

Academic RVU Survey

Q1 - Please rate each of the following residency activities based on required effort: **Minimal amount of effort:** " I don't need to create anything new, but I do need to be present for a short amount of time." **Some amount of effort:** "I need to deliver a small amount of new content or be present for a chunk of time, but I am not extending myself." **Good amount of effort:** "This requires new material and a good amount of time commitment." **Most amount of effort:** "This activity takes up all my time and I have no time for anything else."

		Minimum	Maximum	Mean	Std Deviation	Variance	Count
1	Residency module leader (non-APD responsible for leading a month long conference module, which includes planning, delivering and assigning all content for the whole month)	1.00	4.00	3.33	0.77	0.59	49
2	Morning report attendance (attendance at resident education session after signout is completed at BH (occurs on M, T, Th, Fri 8:15-9am)	1.00	3.00	1.22	0.46	0.21	49
3	Lecture at resident conference (prepare and present an original faculty lecture)	1.00	4.00	3.00	0.53	0.29	49
4	Journal club moderator at resident conference (leader of a journal club discussion)	1.00	4.00	2.37	0.63	0.40	49
5	Preceptor morbidity and mortality conference (primary mentor of M&M session)	1.00	4.00	2.27	0.69	0.48	49
6	Preceptor/Participant Sonolympics (moderator of 4 hour US education competition)	1.00	4.00	2.18	0.80	0.64	49
7	Preceptor/Participant EM Olympics (moderator of 4 hour EM education competition)	1.00	4.00	2.12	0.82	0.68	49
8	Faculty participant/mentor CPC (primary mentor and participant in the clinical pathologic case conference competition)	1.00	4.00	2.86	0.78	0.61	49

		Minimum	Maximum	Mean	Std Deviation	Variance	Count
9	EM Foundations Curriculum Faculty Facilitator (leader small group sessions with residents guiding them through oral boards type cases and group discussions)	1.00	4.00	2.18	0.80	0.64	49
10	Primary mentor on resident lecture (review, edit and provide feedback on content of original lecture)	1.00	3.00	2.02	0.65	0.43	49
11	Instructor at resident procedure, simulation or multi-modal workshop (teach at US, simulation, procedure workshops requiring 4-hour time commitment)	1.00	4.00	2.24	0.69	0.47	49
12	Small groups facilitator at resident conference (leader of any small group session during Wednesday conference)	1.00	3.00	2.02	0.68	0.47	49
13	Leader Scholarly Academy (create monthly longitudinal curriculum, invite speakers, organize workshops and lectures)	1.00	4.00	3.27	0.80	0.64	49
14	Attendance at Scholarly Academy (attend SA meetings as participant)	1.00	3.00	1.35	0.52	0.27	49
15	Leader in-situ Simulation Session (leading faculty member in resuscitation room during in-situ session)	1.00	4.00	2.22	0.65	0.42	49
16	Member of Residency Program Evaluation Committee (PEC) (non-APD member of the committee responsible for evaluating the residency, generating a report to the GME office and developing new initiatives to improve the residency program)	1.00	4.00	2.47	0.93	0.86	49
17	Mentor Scholarly Project (mentor on a resident project that has real outcomes (ie published product, presentation at a conference, new initiative in the department or other actvities)	1.00	4.00	2.86	0.78	0.61	49
18	Member of Clinical Competency Committee (CCC) (non-APD member of the committee responsible for reviewing residents' competency based on milestones)	1.00	3.00	2.27	0.69	0.48	49
19	Residency interviewer (non-APD participant in residency interview dates)	1.00	4.00	2.39	0.88	0.77	49
20	Participation in Standardized Direct Observation Assessment Tool (SDOT) (observe and provide feedback to a resident providing patient care while on shift)	1.00	3.00	1.61	0.66	0.44	49
21	Participation in oral boards preparation (preceptor of oral boards cases requiring 4 hours of faculty commitment)	1.00	3.00	1.98	0.59	0.35	49

		Minimal amount of effort		Some amount of effort		Good amount of effort		Most amount of effort		Total
1	Residency module leader (non-APD responsible for leading a month long conference module, which includes planning, delivering and assigning all content for the whole month)	4.08%	2	6.12%	3	42.86%	21	46.94%	23	49
2	Morning report attendance (attendance at resident education session after signout is completed at BH (occurs on M, T, Th, Fri 8:15-9am)	79.59%	39	18.37%	9	2.04%	1	0.00%	0	49
3	Lecture at resident conference (prepare and present an original faculty lecture)	2.04%	1	8.16%	4	77.55%	38	12.24%	6	49
4	Journal club moderator at resident conference (leader of a journal club discussion)	6.12%	3	53.06%	26	38.78%	19	2.04%	1	49
5	Preceptor morbidity and mortality conference (primary mentor of M&M session)	10.20%	5	57.14%	28	28.57%	14	4.08%	2	49
6	Preceptor/Participant Sonolympics (moderator of 4 hour US education competition)	18.37%	9	51.02%	25	24.49%	12	6.12%	3	49
7	Preceptor/Participant EM Olympics (moderator of 4 hour EM education competition)	22.45%	11	48.98%	24	22.45%	11	6.12%	3	49
8	Faculty participant/mentor CPC (primary mentor and participant in the clinical pathologic case conference competition)	4.08%	2	26.53%	13	48.98%	24	20.41%	10	49
9	EM Foundations Curriculum Faculty Facilitator (leader small group sessions with residents guiding them through oral boards type cases and group discussions)	18.37%	9	51.02%	25	24.49%	12	6.12%	3	49
10	Primary mentor on resident lecture (review, edit and provide feedback on content of original lecture)	20.41%	10	57.14%	28	22.45%	11	0.00%	0	49
11	Instructor at resident procedure, simulation or multi-modal workshop (teach at US, simulation, procedure workshops requiring 4-hour time commitment)	10.20%	5	59.18%	29	26.53%	13	4.08%	2	49
12	Small groups facilitator at resident conference (leader of any small group session during Wednesday conference)	22.45%	11	53.06%	26	24.49%	12	0.00%	0	49
13	Leader Scholarly Academy (create monthly longitudinal curriculum, invite speakers, organize workshops and lectures)	4.08%	2	10.20%	5	40.82%	20	44.90%	22	49
14	Attendance at Scholarly Academy (attend SA meetings as participant)	67.35%	33	30.61%	15	2.04%	1	0.00%	0	49
15	Leader in-situ Simulation Session (leading faculty member in resuscitation room during in-situ session)	8.16%	4	65.31%	32	22.45%	11	4.08%	2	49
16	Member of Residency Program Evaluation Committee (PEC) (non-APD member of the committee responsible for evaluating the residency, generating a report to the GME office and developing new initiatives to improve the residency program)	16.33%	8	34.69%	17	34.69%	17	14.29%	7	49
17	Mentor Scholarly Project (mentor on a resident project that has real outcomes (ie published product, presentation at a conference, new initiative in the department or other actvities)	6.12%	3	20.41%	10	55.10%	27	18.37%	9	49

		Minimal amount of effort		Some amount of effort		Good amount of effort		Most amount of effort		Total
18	Member of Clinical Competency Committee (CCC) (non-APD member of the committee responsible for reviewing residents' competency based on milestones)	14.29%	7	44.90%	22	40.82%	20	0.00%	0	49
19	Residency interviewer (non-APD participant in residency interview dates)	16.33%	8	38.78%	19	34.69%	17	10.20%	5	49
20	Participation in Standardized Direct Observation Assessment Tool (SDOT) (observe and provide feedback to a resident providing patient care while on shift)	48.98%	24	40.82%	20	10.20%	5	0.00%	0	49
21	Participation in oral boards preparation (preceptor of oral boards cases requiring 4 hours of faculty commitment)	18.37%	9	65.31%	32	16.33%	8	0.00%	0	49

Q2 - Please rate each of the following medical student activities based on required effort:

Minimal amount of effort: " I don't need to create anything new, but I do need to be present for a short amount of time." **Some amount of effort:** "I need to deliver a small amount of new content or be present for a chunk of time, but I am not extending myself." **Good amount of effort:** "This requires new material and a good amount of time commitment." **Most amount of effort:** "This activity takes up all my time and I have no time for anything else."

		Minimum	Maximum	Mean	Std Deviation	Variance	Count
1	Course director of medical student selective course (organize and deliver content, as well as evaluate student for selective grades)	1.00	4.00	3.57	0.70	0.49	49
2	Course director of medical student elective course (organize and deliver content for elective course)	1.00	4.00	3.49	0.70	0.49	49
3	Medical student case session for EM selective or elective (1-hour session (UME provides the teaching materials/case) to students during orientation or Wednesday conference)	1.00	4.00	1.76	0.77	0.59	49
4	Primary medical student faculty mentor (assigned mentor to a student, requiring several meetings/year, working together for 4 clinical shifts and providing a summative assessment at the end of the rotation)	1.00	3.00	2.06	0.65	0.42	49
5	Primary URiM Summer Fellowship Student faculty mentor (assigned faculty mentor to an EM URiM Summer Fellowship student, requiring working together for 5-10 4-hour clinical shifts and participation in mentoring meetings)	1.00	4.00	2.29	0.76	0.57	49
6	Participant URiM Summer Fellowship(create content and lead a session with the EM URiM Summer Fellowship students, requiring faculty commitment of 1-2 hours/session)	1.00	4.00	2.24	0.74	0.55	49
7	Instructor in student simulation or workshop sessions (1-2 hour long workshops for students in ultrasound, trauma, critical care, ACS, or ACLS)	1.00	3.00	1.88	0.59	0.35	49
8	Instructor Transition to Residency Course (NYUSoM course, helping students transition to residency, requiring faculty commitment in 4 hour blocks of time)	1.00	3.00	2.16	0.58	0.34	49
9	Instructor Inter-clerkship Intensive (ICI) Courses(NYUSoM course, teaching curriculum not well-covered in the clerkships, requiring faculty commitment in 4 hour blocks of time)	1.00	4.00	2.24	0.66	0.43	49
10	Instructor in Practice of Medicine (participate in bedside teaching sessions for students focusing on history and physical exam skills, requiring faculty commitment in 3.5 hour blocks of time)	1.00	4.00	2.24	0.72	0.51	49

		Minimum	Maximum	Mean	Std Deviation	Variance	Count
11	Preceptor for the Patient Longitudinal Ambulatory Clinical Experience (PLACE) (participate in sessions that allow students to practice clinical skills taught in the Practice of Medicine course; each session requires 2 hours or longer and a total faculty commitment of 8 hours per student)	1.00	4.00	2.43	0.73	0.53	49
12	Lecture in Basic Science Course (provide a student lecture of pre-clinical content, time alloted by UME)	1.00	4.00	2.59	0.81	0.65	49
13	Participation in Emergency Medicine Interest Group (participate in 2-3 hour long sessions teaching students procedures, EKGs, ultrasound, and other topics)	1.00	3.00	1.86	0.61	0.37	49
14	Lecture in Medical School Course for elective or selective (provide a student lecture for a selective or elective course, time alloted by UME)	1.00	4.00	2.37	0.77	0.60	49
15	Instructor medical student ultrasound workshops (teach student US workshops throughout the year with a faculty commitment of 4-hour blocks)	1.00	3.00	2.20	0.67	0.45	49
16	Medical school interviews (participate in college student interviews for the NYUSoM)	1.00	3.00	2.00	0.70	0.49	49
17	Preceptor of Medical Student Scholarly, Research Concentration Project or Research Elective (mentor a student for a 12-week long scholarly or research project)	1.00	4.00	2.61	0.72	0.52	49
18	Mentor of Medical Student International Health Program (mentor a student on an IRB approved international health project as the faculty principal investigator, ensuring its academic quality and serving as point of contact with the IHP program)	1.00	4.00	2.65	0.87	0.76	49
19	Preceptor of medical student ultrasound OSCE (conduct OSCE to assess students at end of their US elective requiring a faculty committment of 1 hour/student)	1.00	3.00	1.80	0.61	0.37	49

		Minimal amount of effort		Some amount of effort		Good amount of effort		Most amount of effort		Total
1	Course director of medical student selective course (organize and deliver content, as well as evaluate student for selective grades)	4.08%	2	0.00%	0	30.61%	15	65.31%	32	49
2	Course director of medical student elective course (organize and deliver content for elective course)	4.08%	2	0.00%	0	38.78%	19	57.14%	28	49
3	Medical student case session for EM selective or elective (1-hour session (UME provides the teaching materials/case) to students during orientation or Wednesday conference)	42.86%	21	40.82%	20	14.29%	7	2.04%	1	49

		Minimal amount of effort		Some amount of effort		Good amount of effort		Most amount of effort		Total
4	Primary medical student faculty mentor (assigned mentor to a student, requiring several meetings/year, working together for 4 clinical shifts and providing a summative assessment at the end of the rotation)	18.37%	9	57.14%	28	24.49%	12	0.00%	0	49
5	Primary URiM Summer Fellowship Student faculty mentor (assigned faculty mentor to an EM URiM Summer Fellowship student, requiring working together for 5-10 4-hour clinical shifts and participation in mentoring meetings)	14.29%	7	46.94%	23	34.69%	17	4.08%	2	49
6	Participant URiM Summer Fellowship(create content and lead a session with the EM URiM Summer Fellowship students, requiring faculty commitment of 1-2 hours/session)	14.29%	7	51.02%	25	30.61%	15	4.08%	2	49
7	Instructor in student simulation or workshop sessions (1-2 hour long workshops for students in ultrasound, trauma, critical care, ACS, or ACLS)	24.49%	12	63.27%	31	12.24%	6	0.00%	0	49
8	Instructor Transition to Residency Course (NYUSoM course, helping students transition to residency, requiring faculty commitment in 4 hour blocks of time)	10.20%	5	63.27%	31	26.53%	13	0.00%	0	49
9	Instructor Inter-clerkship Intensive (ICI) Courses(NYUSoM course, teaching curriculum not well-covered in the clerkships, requiring faculty commitment in 4 hour blocks of time)	10.20%	5	57.14%	28	30.61%	15	2.04%	1	49
10	Instructor in Practice of Medicine (participate in bedside teaching sessions for students focusing on history and physical exam skills, requiring faculty commitment in 3.5 hour blocks of time)	12.24%	6	55.10%	27	28.57%	14	4.08%	2	49
11	Preceptor for the Patient Longitudinal Ambulatory Clinical Experience (PLACE) (participate in sessions that allow students to practice clinical skills taught in the Practice of Medicine course; each session requires 2 hours or longer and a total faculty commitment of 8 hours per student)	8.16%	4	46.94%	23	38.78%	19	6.12%	3	49
12	Lecture in Basic Science Course (provide a student lecture of pre-clinical content, time alloted by UME)	10.20%	5	30.61%	15	48.98%	24	10.20%	5	49
13	Participation in Emergency Medicine Interest Group (participate in 2-3 hour long sessions teaching students procedures, EKGs, ultrasound, and other topics)	26.53%	13	61.22%	30	12.24%	6	0.00%	0	49
14	Lecture in Medical School Course for elective or selective (provide a student lecture for a selective or elective course, time alloted by UME)	16.33%	8	32.65%	16	48.98%	24	2.04%	1	49
15	Instructor medical student ultrasound workshops (teach student US workshops throughout the year with a faculty commitment of 4-hour blocks)	14.29%	7	51.02%	25	34.69%	17	0.00%	0	49
16	Medical school interviews (participate in college student interviews for the NYUSoM)	24.49%	12	51.02%	25	24.49%	12	0.00%	0	49
17	Preceptor of Medical Student Scholarly, Research Concentration Project or Research Elective (mentor a student for a 12-week long scholarly or research project)	8.16%	4	28.57%	14	57.14%	28	6.12%	3	49

		Minimal amount of effort		Some amount of effort		Good amount of effort		Most amount of effort		Total
18	Mentor of Medical Student International Health Program (mentor a student on an IRB approved international health project as the faculty principal investigator, ensuring its academic quality and serving as point of contact with the IHP program)	12.24%	6	24.49%	12	48.98%	24	14.29%	7	49
19	Preceptor of medical student ultrasound OSCE (conduct OSCE to assess students at end of their US elective requiring a faculty committment of 1 hour/student)	30.61%	15	59.18%	29	10.20%	5	0.00%	0	49

Q3 - Please rate each of the following other educational activities based on required effort: **Minimal amount of effort:** " I don't need to create anything new, but I do need to be present for a short amount of time." **Some amount of effort:** "I need to deliver a small amount of new content or be present for a chunk of time, but I am not extending myself." **Good amount of effort:** "This requires new material and a good amount of time commitment." **Most amount of effort:** "This activity takes up all my time and I have no time for anything else."

		Minimum	Maximum	Mean	Std Deviation	Variance	Count
1	Leader at faculty development session (plan, coordinate and run a faculty development session)	1.00	4.00	2.94	0.79	0.63	49
2	Attendance at faculty development session (attendance at any faculty development session)	1.00	3.00	1.29	0.49	0.24	49
3	Lecture at PEM conference (prepare and present an original faculty lecture)	1.00	4.00	3.00	0.57	0.33	49
4	PEM conference journal club moderator (lead a journal club discussion)	1.00	4.00	2.27	0.60	0.36	49
5	PEM journal update presenter (present the journal article update chosen for PEM conference)	1.00	4.00	2.27	0.60	0.36	49
6	PEM joint conference liaison (arrange and lead joint conferences with Radiology, Cardiology, Peds Surgery and PICU)	1.00	3.00	2.43	0.64	0.41	49
7	Instructor at PEM procedure or simulation workshop (teach at US, simulation, procedure workshops requiring 1-hour time commitment per month)	1.00	3.00	2.10	0.61	0.38	49

		Minimum	Maximum	Mean	Std Deviation	Variance	Count
8	Participation in EM/PEM conference (preceptor of PGY4 pediatric case presented during morning report)	1.00	3.00	1.59	0.67	0.45	49
9	PALS instructor (instructor in course requiring 4 hour faculty time commitment)	1.00	4.00	1.98	0.65	0.43	49
10	Attendance at Education Journal Club (participate in discussion of relevant education journal articles at a 2 hour session)	1.00	3.00	1.47	0.58	0.33	49
11	Attendance at PSQI Journal Club (participate in discussion of relevant quality and safety journal articles at a 1 hour session)	1.00	3.00	1.41	0.57	0.32	49
12	Attendance at Toxicology Journal Club (participate in discussion of relevant toxicology journal articles at a 1.5 - 2 hour session)	1.00	3.00	1.43	0.57	0.33	49
13	Preceptor of Toxicology fellow rounds (fellows present last 24-hour call of critical cases and faculty teach/discuss requiring 1.5 hour commitment)	1.00	4.00	2.02	0.71	0.51	49
14	Preceptor of Toxicology bedside rounds (faculty leader and teacher of bedside consult rounds)	1.00	4.00	2.08	0.75	0.56	49
15	Attendance at Toxicology Consultants' Conference (monthly 2 hour conference discussing prominent Toxicology cases)	1.00	3.00	1.31	0.54	0.29	49
16	Lecture at Toxicology Rotators Conference (1 hour lecture given by Tox faculty)	1.00	4.00	2.39	0.69	0.48	49
17	Partcipate in NYCPCC afternoon rounds (2-3 hour didactic/group discussion teaching rounds)	1.00	4.00	1.94	0.79	0.63	49
18	Member of national/regional education committee (participate on a committee with an education focus at a national or regional level)	1.00	3.00	2.27	0.69	0.48	49
19	Chair of national/regional education committee (leader of a committee with an education focus at a national or regional level)	1.00	4.00	3.12	0.72	0.52	49
20	Member of NYUSoM educational committee (participate on a NYUSoM committee with an education focus, ie curriculum committee, mentorship committee or others)	1.00	4.00	2.37	0.80	0.64	49
21	Attendance at Ultrasound Conference (participate in journal club, image review, didactics and resident case presentations, requiring 2-3 hour faculty commitment)	1.00	3.00	1.49	0.61	0.37	49
22	Ultrasound scanning shifts with residents (participate in hands-on scanning shifts with residents requiring 2-3 hour faculty commitment)	1.00	4.00	1.96	0.60	0.37	49
23	Ultrasound scanning shifts with medical students (participate in hands-on scanning shifts with students requiring 2-3 hour faculty commitment)	1.00	4.00	1.96	0.60	0.37	49

		Minimum	Maximum	Mean	Std Deviation	Variance	Count
24	Lecture to other NYU residents, faculty, students or staff (invited lecturer to another department within NYUSoM)	1.00	4.00	2.57	0.70	0.49	49
25	Grand rounds lecture - external institution (official Grand Rounds invite to lecture at an outside institution)	1.00	4.00	3.16	0.71	0.50	49
26	Grand rounds lecture - internal at NYU (official Grand Rounds invite to lecture for the Department of EM or another department within NYUSoM)	1.00	4.00	3.08	0.78	0.61	49
27	Outside lecture or teaching session at another teaching institution (invite to lecture or teach at an outside institution - not Grand Rounds)	1.00	4.00	2.98	0.71	0.51	49
28	Lecture at international, national or regional meeting (lecture at a meeting, such as ACEP, SAEM, CORD or others)	1.00	4.00	3.24	0.72	0.51	49
29	Lecture at fellow core curriculum session (create content and lead lecture or session for joint departmental fellowship curriculum, requiring 1 hour faculty commitment)	1.00	4.00	2.65	0.69	0.47	49
30	Content creator and/or editor of educational site/blog/podcast (such as CoreEM, MD Calc or others)	1.00	4.00	2.92	0.85	0.73	49
31	PEP talks to students (participate in school student talks/lectures through PEP, requiring 2-3 hour faculty commitment)	1.00	4.00	2.04	0.67	0.45	49
32	Instructor ATLS (faculty instructor for house staff 2-day ATLS course)	1.00	4.00	2.14	0.81	0.65	49
33	Participation in First Night on Call for Interns (teach at GME event preparing interns for patient care, requiring faculty commitment in blocks of 2-4 hours)	1.00	4.00	2.14	0.67	0.45	49

		Minimal amount of effort		Some amount of effort		Good amount of effort		Most amount of effort		Total
1	Leader at faculty development session (plan, coordinate and run a faculty development session)	6.12%	3	16.33%	8	55.10%	27	22.45%	11	49
2	Attendance at faculty development session (attendance at any faculty development session)	73.47%	36	24.49%	12	2.04%	1	0.00%	0	49
3	Lecture at PEM conference (prepare and present an original faculty lecture)	2.04%	1	10.20%	5	73.47%	36	14.29%	7	49
4	PEM conference journal club moderator (lead a journal club discussion)	6.12%	3	63.27%	31	28.57%	14	2.04%	1	49
5	PEM journal update presenter (present the journal article update chosen for PEM conference)	6.12%	3	63.27%	31	28.57%	14	2.04%	1	49

		Minimal amount of effort		Some amount of effort		Good amount of effort		Most amount of effort		Total
6	PEM joint conference liaison (arrange and lead joint conferences with Radiology, Cardiology, Peds Surgery and PICU)	8.16%	4	40.82%	20	51.02%	25	0.00%	0	49
7	Instructor at PEM procedure or simulation workshop (teach at US, simulation, procedure workshops requiring 1-hour time commitment per month)	14.29%	7	61.22%	30	24.49%	12	0.00%	0	49
8	Participation in EM/PEM conference (preceptor of PGY4 pediatric case presented during morning report)	51.02%	25	38.78%	19	10.20%	5	0.00%	0	49
9	PALS instructor (instructor in course requiring 4 hour faculty time commitment)	20.41%	10	63.27%	31	14.29%	7	2.04%	1	49
10	Attendance at Education Journal Club (participate in discussion of relevant education journal articles at a 2 hour session)	57.14%	28	38.78%	19	4.08%	2	0.00%	0	49
11	Attendance at PSQI Journal Club (participate in discussion of relevant quality and safety journal articles at a 1 hour session)	63.27%	31	32.65%	16	4.08%	2	0.00%	0	49
12	Attendance at Toxicology Journal Club (participate in discussion of relevant toxicology journal articles at a 1.5 - 2 hour session)	61.22%	30	34.69%	17	4.08%	2	0.00%	0	49
13	Preceptor of Toxicology fellow rounds (fellows present last 24-hour call of critical cases and faculty teach/discuss requiring 1.5 hour commitment)	20.41%	10	61.22%	30	14.29%	7	4.08%	2	49
14	Preceptor of Toxicology bedside rounds (faculty leader and teacher of bedside consult rounds)	20.41%	10	55.10%	27	20.41%	10	4.08%	2	49
15	Attendance at Toxicology Consultants' Conference (monthly 2 hour conference discussing prominent Toxicology cases)	73.47%	36	22.45%	11	4.08%	2	0.00%	0	49
16	Lecture at Toxicology Rotators Conference (1 hour lecture given by Tox faculty)	10.20%	5	42.86%	21	44.90%	22	2.04%	1	49
17	Partcipate in NYCPCC afternoon rounds (2-3 hour didactic/group discussion teaching rounds)	32.65%	16	42.86%	21	22.45%	11	2.04%	1	49
18	Member of national/regional education committee (participate on a committee with an education focus at a national or regional level)	14.29%	7	44.90%	22	40.82%	20	0.00%	0	49
19	Chair of national/regional education committee (leader of a committee with an education focus at a national or regional level)	4.08%	2	8.16%	4	59.18%	29	28.57%	14	49
20	Member of NYUSoM educational committee (participate on a NYUSoM committee with an education focus, ie curriculum committee, mentorship committee or others)	12.24%	6	46.94%	23	32.65%	16	8.16%	4	49
21	Attendance at Ultrasound Conference (participate in journal club, image review, didactics and resident case presentations, requiring 2-3 hour faculty commitment)	57.14%	28	36.73%	18	6.12%	3	0.00%	0	49
22	Ultrasound scanning shifts with residents (participate in hands-on scanning shifts with residents requiring 2-3 hour faculty commitment)	18.37%	9	69.39%	34	10.20%	5	2.04%	1	49

		Minimal amount of effort		Some amount of effort		Good amount of effort		Most amount of effort		Total
23	Ultrasound scanning shifts with medical students (participate in hands-on scanning shifts with students requiring 2-3 hour faculty commitment)	18.37%	9	69.39%	34	10.20%	5	2.04%	1	49
24	Lecture to other NYU residents, faculty, students or staff (invited lecturer to another department within NYUSoM)	8.16%	4	30.61%	15	57.14%	28	4.08%	2	49
25	Grand rounds lecture - external institution (official Grand Rounds invite to lecture at an outside institution)	4.08%	2	6.12%	3	59.18%	29	30.61%	15	49
26	Grand rounds lecture - internal at NYU (official Grand Rounds invite to lecture for the Department of EM or another department within NYUSoM)	6.12%	3	8.16%	4	57.14%	28	28.57%	14	49
27	Outside lecture or teaching session at another teaching institution (invite to lecture or teach at an outside institution - not Grand Rounds)	4.08%	2	14.29%	7	61.22%	30	20.41%	10	49
28	Lecture at international, national or regional meeting (lecture at a meeting, such as ACEP, SAEM, CORD or others)	4.08%	2	4.08%	2	55.10%	27	36.73%	18	49
29	Lecture at fellow core curriculum session (create content and lead lecture or session for joint departmental fellowship curriculum, requiring 1 hour faculty commitment)	6.12%	3	28.57%	14	59.18%	29	6.12%	3	49
30	Content creator and/or editor of educational site/blog/podcast (such as CoreEM, MD Calc or others)	6.12%	3	22.45%	11	44.90%	22	26.53%	13	49
31	PEP talks to students (participate in school student talks/lectures through PEP, requiring 2-3 hour faculty commitment)	18.37%	9	61.22%	30	18.37%	9	2.04%	1	49
32	Instructor ATLS (faculty instructor for house staff 2-day ATLS course)	20.41%	10	51.02%	25	22.45%	11	6.12%	3	49
33	Participation in First Night on Call for Interns (teach at GME event preparing interns for patient care, requiring faculty commitment in blocks of 2-4 hours)	12.24%	6	65.31%	32	18.37%	9	4.08%	2	49

Q4 - Please rate each of the following scholarly activities based on required effort: **Minimal amount of effort:** " I don't need to create anything new, but I do need to be present for a short amount of time." **Some amount of effort:** "I need to deliver a small amount of new content or be present for a chunk of time, but I am not extending myself." **Good amount of effort:** "This requires new material and a good amount of time commitment." Most amount of effort: "This activity takes up all my time and I have no time for anything else."

		Minimum	Maximum	Mean	Std Deviation	Variance	Count
1	Principal Investigator (PI) on federal grant	1.00	4.00	3.80	0.64	0.41	49
2	Co-Investigator on federal grant	1.00	4.00	3.27	0.72	0.52	49
3	Principal Investigator (PI) on foundation grant	1.00	4.00	3.71	0.67	0.45	49
4	Co-Investigator on foundation grant	1.00	4.00	3.18	0.72	0.52	49
5	Principal Investigator (PI) on industry grant	1.00	4.00	3.65	0.69	0.47	49
6	Principal Investigator (PI) on internal NYUSoM grant	1.00	4.00	3.49	0.79	0.62	49
7	Principal Investigator (PI) on internal Department of EM grant	1.00	4.00	3.43	0.81	0.65	49
8	First author peer-reviewed research manuscript (first author on publication with PMID#)	1.00	4.00	3.55	0.70	0.49	49

		Minimum	Maximum	Mean	Std Deviation	Variance	Count
9	Last author peer-reviewed research manuscript (last author on= publication with PMID#)	1.00	4.00	2.78	0.89	0.79	49
10	Co-author peer-reviewed research manuscript (co-author on= publication with PMID#)	1.00	4.00	2.73	0.69	0.48	49
11	First author non-research manuscript (first author on publication= without PMID#)	1.00	4.00	3.16	0.68	0.46	49
12	Last author non-research manuscript (last author on publication= without PMID#)	1.00	4.00	2.53	0.81	0.66	49
13	Co-author non-research manuscript (co-author on publication= without PMID#)	1.00	4.00	2.53	0.70	0.49	49
14	First Author on Case report	1.00	4.00	2.53	0.64	0.41	49
15	Commentary/letter to editor (any author)	1.00	4.00	2.20	0.70	0.49	49
16	Primary textbook editor (main editor)	1.00	4.00	3.53	0.79	0.62	49
17	Textbook chapter (author)	1.00	4.00	3.27	0.72	0.52	49
18	Abstract presenter national meeting (main author and presenter= at a national meeting)	1.00	4.00	2.80	0.64	0.41	49
19	Abstract presenter regional/local meeting (main author and= presenter at a regional or local meeting)	1.00	4.00	2.71	0.67	0.45	49
20	Co-author abstract national meeting (co-author, but not= presenter at a national meeting)	1.00	3.00	2.33	0.71	0.50	49
21	Co-author abstract regional meeting (co-author, but not= presenter at regional meeting)	1.00	4.00	2.31	0.77	0.59	49
22	Journal editor (on editorial board of peer reviewed journal)	1.00	4.00	3.14	0.78	0.61	49
23	Journal reviewer (manuscript reviewer for peer reviewed journal)	1.00	4.00	2.57	0.76	0.57	49

		Minimal amount of effort		Some amount of effort		Good amount of effort		Most amount of effort		Total
1	Principal Investigator (PI) on federal grant	4.08%	2	0.00%	0	8.16%	4	87.76%	43	49

		Minimal amount of effort		Some amount of effort		Good amount of effort		Most amount of effort		Total
2	Co-Investigator on federal grant	4.08%	2	4.08%	2	53.06%	26	38.78%	19	49
3	Principal Investigator (PI) on foundation grant	4.08%	2	0.00%	0	16.33%	8	79.59%	39	49
4	Co-Investigator on foundation grant	4.08%	2	6.12%	3	57.14%	28	32.65%	16	49
5	Principal Investigator (PI) on industry grant	4.08%	2	0.00%	0	22.45%	11	73.47%	36	49
6	Principal Investigator (PI) on internal NYUSoM grant	4.08%	2	6.12%	3	26.53%	13	63.27%	31	49
7	Principal Investigator (PI) on internal Department of EM grant	4.08%	2	8.16%	4	28.57%	14	59.18%	29	49
8	First author peer-reviewed research manuscript (first author on publication with PMID#)	4.08%	2	0.00%	0	32.65%	16	63.27%	31	49
9	Last author peer-reviewed research manuscript (last author on publication with PMID#)	10.20%	5	22.45%	11	46.94%	23	20.41%	10	49
10	Co-author peer-reviewed research manuscript (co-author on publication with PMID#)	4.08%	2	28.57%	14	57.14%	28	10.20%	5	49
11	First author non-research manuscript (first author on publication without PMID#)	4.08%	2	4.08%	2	63.27%	31	28.57%	14	49
12	Last author non-research manuscript (last author on publication without PMID#)	10.20%	5	36.73%	18	42.86%	21	10.20%	5	49
13	Co-author non-research manuscript (co-author on publication without PMID#)	6.12%	3	40.82%	20	46.94%	23	6.12%	3	49
14	Author on Case report (any author)	2.04%	1	48.98%	24	42.86%	21	6.12%	3	49
15	Commentary/letter to editor (any author)	12.24%	6	59.18%	29	24.49%	12	4.08%	2	49
16	Primary textbook editor (main editor)	4.08%	2	6.12%	3	22.45%	11	67.35%	33	49
17	Textbook chapter (author)	4.08%	2	4.08%	2	53.06%	26	38.78%	19	49
18	Abstract presenter national meeting (main author and presenter at a national meeting)	4.08%	2	20.41%	10	67.35%	33	8.16%	4	49
19	Abstract presenter regional/local meeting (main author and presenter at a regional or local meeting)	4.08%	2	28.57%	14	59.18%	29	8.16%	4	49
20	Co-author abstract national meeting (co-author, but not presenter at a national meeting)	14.29%	7	40.82%	20	44.90%	22	0.00%	0	49
21	Co-author abstract regional meeting (co-author, but not presenter at regional meeting)	14.29%	7	42.86%	21	38.78%	19	4.08%	2	49
22	Journal editor (on editorial board of peer reviewed journal)	4.08%	2	12.24%	6	48.98%	24	34.69%	17	49
23	Journal reviewer (manuscript reviewer for peer reviewed journal)	8.16%	4	34.69%	17	48.98%	24	8.16%	4	49
